# Divergent Rehabilitation Targets in Pediatric Cerebral Palsy: Asymmetry-Bounded Versus Symmetry-Disrupting Strategies in Hemiplegic and Diplegic Gait

**DOI:** 10.3390/jfmk11040376

**Published:** 2026-09-22

**Authors:** Teodora Dominteanu, Marius Dumitru Dima, Amelia Elena Stan, Andreea Voinea

**Affiliations:** 1 Department of Physical Education and Sport, Faculty of Agrifood and Environmental, Bucharest University of Economic Studies, 010374 Bucharest, Romania; 2 Faculty of Physical Education and Sport, Romanian-American University, Exhibition Boulevard No. 1B, Sector 1, 012101 Bucharest, Romania; marius.dima@rau.ro (M.D.D.); amelia.stan@rau.ro (A.E.S.)

**Keywords:** cerebral palsy, hemiplegia, diplegia, gait asymmetry, gait symmetry index, motor control, neuromuscular control, postural control, rehabilitation strategies, developmental trajectory

## Abstract

Rehabilitation for gait asymmetry in cerebral palsy (CP) commonly treats improvements in gait symmetry indices as a subtype-general marker of therapeutic success. Building on a trajectory-divergence model developed in a companion review, we propose that hemiplegic (unilateral spastic) and diplegic (bilateral spastic) CP—using Surveillance of Cerebral Palsy in Europe (SCPE) nomenclature—represent opposing developmental deviations from the normative gait symmetry trajectory—persistent, actively regulated compensatory asymmetry in hemiplegia versus pathological rigid symmetry in diplegia—such that a single, shared correction target is conceptually misaligned for at least one subtype. This review critically evaluates, through a narrative synthesis of literature published between 2010 and 2026, whether rehabilitation strategies for hemiplegic and diplegic CP should be differentiated according to this model rather than converging on gait symmetry restoration. A structured narrative review was conducted following a Population–Concept–Context (PCC) framework, drawing on 70 unique sources originally identified through 21 structured search queries organized into ten thematic groups, supplemented by four additional sources incorporated during peer review. In hemiplegic CP, compensatory asymmetry is mechanistically and energetically dissociable from correction, but its magnitude, not its presence, predicts balance and locomotor outcomes, bounding rather than eliminating the case for conservation. In diplegic CP, restoring adaptive motor variability is supported by convergent evidence but is conditional on confirming genuine bilateral rigidity, since a meaningful subset of diagnosed diplegic children presents with clinically significant inter-limb asymmetry. A provisional Divergent Rehabilitation Response Framework (DRRF) organizes four domains of subtype-specific therapeutic congruence: counter-evidence, such as correction-beneficial cases, inconsistent functional transfer, evidentiary concentration, and long-term musculoskeletal risk signals, are directly addressed. Differentiated magnitude- and subtype-conditional rehabilitation targets, rather than universal symmetrization, are proposed for hemiplegic and diplegic CP, alongside an explicit research agenda.

## 1. Introduction

Typical gait develops toward a stable, reliable pattern of bilateral symmetry over childhood, with asymmetry magnitude decreasing and inter-session reliability increasing as the neuromuscular system matures, a normative trajectory recently characterized in quantitative detail by a systematic review and synthesis of gait symmetry reliability and asymmetry magnitude in typically developing children [1]. Cerebral palsy (CP), the most common cause of childhood physical disability, disrupts this trajectory; however, not in a single, uniform direction. Hemiplegic CP, in which motor deficits predominate unilaterally, and diplegic (bilateral spastic) CP, in which both lower limbs are affected, are treated, in a substantial portion of the intervention literature, as variations on a shared underlying problem—“asymmetric” or “abnormal” gait. Both are commonly corrected toward the same normative target of greater bilateral symmetry. This tendency is illustrated by several intervention studies discussed in Section 6.1, which report gait symmetry index improvement as a primary marker of therapeutic success regardless of subtype (e.g., [2]). We do not claim, however, that this reflects a universally codified clinical guideline.

Our companion narrative review [3] proposed a trajectory-divergence model to describe why this shared target may be conceptually mistaken: hemiplegic and diplegic CP represent opposing deviations from the normative developmental symmetry curve, not two variants of the same deviation. In hemiplegic CP, gait asymmetry tends to persist and, in important respects, to be actively regulated—a compensatory adaptation of a nervous system that retains independent control over each limb, rather than a simple, uncontrolled consequence of unilateral damage. In diplegic CP, by contrast, the more characteristic abnormality is not asymmetry but an excessively rigid, over-coupled bilateral coordination pattern—a pathological form of symmetry, reflecting a loss of the adaptive motor variability that typical gait retains even as it becomes reliably bilateral. If this divergence is real, then a rehabilitation paradigm that treats “restoring symmetry” as the shared goal for both subtypes is, at minimum, applying the wrong direction of correction to one of them. Concretely, what is new in the present manuscript relative to [3] is the shift from description to prescription. Reference [3] establishes that hemiplegic and diplegic CP diverge developmentally from the normative symmetry trajectory, but it does not derive rehabilitation targets. It does not propose the magnitude-boundary construct of Section 3.3, nor does it introduce the Divergent Rehabilitation Response Framework of Section 5. It also does not confront the counter-evidence, certainty grading, or clinical recommendations developed in Section 6 and Section 7 of the present review.

The present review makes explicit, at the outset, how this argument’s original claim is transformed: where the companion review [3] developed the trajectory-divergence model primarily as a descriptive and explanatory account of why hemiplegic and diplegic gait diverge developmentally, this review takes that account as its starting premise and asks a prescriptive question: what follows for rehabilitation practice if the divergence is accepted? Specifically, we contest the largely implicit assumption, recurring across a substantial body of current intervention research (Section 6.1 and Section 6.3), that improvement in a gait symmetry index constitutes a valid, subtype-general marker of rehabilitation success. We argue instead that hemiplegic CP calls for a rehabilitation target centered on preventing the escalation of compensatory asymmetry while resisting its reflexive correction. Diplegic CP, by contrast, calls for the restoration of adaptive motor variability rather than further pursuit of an already pathologically rigid symmetric pattern. These are two genuinely different clinical objectives, masked by a shared vocabulary of “asymmetry correction” that has not, to our knowledge, been critically examined in subtype-specific terms in the pediatric CP literature. We note, however, that the broader question of whether reducing gait asymmetry is an appropriate rehabilitation target is not novel in itself. This has been debated for close to a decade in the adult post-stroke neurorehabilitation literature. We draw on that literature here only as an inter-population analogy, providing mechanistic and conceptual context rather than direct evidence concerning children with CP. In this adult, acquired hemiparesis population, Finley and Bastian found that larger foot-placement asymmetries were associated with a lower, not higher, metabolic cost of transport in some individuals with post-stroke hemiparesis, indicating that asymmetry can be metabolically efficient rather than costly [4]. Sánchez and Finley found substantial individual differences in the capacity to reduce asymmetry post-stroke, with only some individuals able to do so without a metabolic penalty [5]. Padmanabhan et al. showed that some individuals post-stroke improve step-length symmetry precisely by walking asymmetrically at other levels of gait organization [6]. These findings concern a different clinical population (adult, acquired, post-stroke hemiparesis) rather than pediatric, developmental CP. They cannot, therefore, be treated as a direct confirmation of the same relationships in hemiplegic or diplegic CP; we present them solely as evidence that the underlying conceptual question has valid precedent elsewhere. We frame the present review’s own contribution as extending this question, by analogy, to pediatric hemiplegic and diplegic CP. Throughout the remainder of the manuscript, our evidence is drawn directly from the pediatric CP population.

Figure 1 summarizes this trajectory divergence model conceptually by plotting the inter-limb asymmetry magnitude against adaptive motor variability. The normative developmental trajectory (companion Symmetry review [1]) moves from high-variability, moderate-asymmetry infant gait toward the low-asymmetry, high-variability “typical mature gait” zone. Hemiplegic CP diverges rightward into a magnitude-bounded compensatory zone, while diplegic CP diverges downward into a low-variability rigidity zone; a minority of diagnosed bilateral spastic CP cases (Section 6.2) fall, unexpectedly, into the compensatory-asymmetry region despite their diagnosis.

Box 1 summarizes the central thesis of this review in brief.

Box 1 **The central thesis, in brief.**

Current cerebral palsy rehabilitation largely treats “restore gait symmetry” as a universal goal. This review argues that this goal is correct for only one of the two subtypes it is applied to. In hemiplegic CP, moderate compensatory asymmetry reflects preserved, independent neural control of each limb and need not be corrected—the therapeutic target is preventing escalation, not enforcing symmetry. In diplegic CP, the more characteristic problem is not asymmetry but pathological rigidity—a loss of the adaptive motor variability that healthy gait retains even as it becomes bilaterally symmetric—and the therapeutic target is restoring variability, not further “correcting” an already-rigid pattern.

This review is structured as follows. Section 2 sets out the literature search strategy, evidence selection criteria (PCC framework), qualitative appraisal approach, and sources of bias, including the concentration of key mechanistic evidence within a small number of research groups, addressed in detail alongside its consequences in Section 6. Section 3 and Section 4 develop the mechanistic, functional, and interventional evidence for the hemiplegic and diplegic arms of the model, respectively, each concluding with an explicit account of the boundary conditions under which the central claim holds. Section 5 introduces the Divergent Rehabilitation Response Framework (DRRF), a provisional heuristic for organizing what would count as evidence of a congruent, subtype-appropriate rehabilitation response rather than a validated clinical instrument. Section 6 confronts the model’s principal counter-evidence directly, including intervention studies in which symmetry-correcting or rigidity-preserving approaches produced functional benefits, subtype heterogeneity, inconsistent functional transfer, evidentiary concentration, and the currently unresolved question of long-term musculoskeletal safety. Section 7 translates this evidence into differentiated, explicitly conditional clinical recommendations, and a specific research agenda. Finally, Section 8 concludes the paper.

## 2. Literature Review Strategy and Scope

### 2.1. Evidence Selection Criteria (PCC Framework)

This review followed the Population–Concept–Context (PCC) framework, consistent with the methodological approach used in the companion review [3].

Population: Children and adolescents with a clinician-confirmed diagnosis of cerebral palsy restricted to the hemiplegic and diplegic subtypes. In accordance with the classification and nomenclature recommended by the Surveillance of Cerebral Palsy in Europe (SCPE), these correspond respectively to unilateral spastic cerebral palsy and bilateral spastic cerebral palsy. For consistency with the terminology used in the great majority of the primary literature cited throughout this review, we retain “hemiplegic” and “diplegic” as the operative terms in the remainder of the manuscript, tables, and figures, on the understanding that they denote unilateral spastic CP and bilateral spastic CP, respectively, as defined by SCPE. Studies addressing other CP subtypes (e.g., dyskinetic and ataxic) were excluded unless a hemiplegic or diplegic subgroup was separately reported. Studies in adults with CP were included only if they addressed long-term consequences (musculoskeletal outcomes, adult mobility trajectories) directly relevant to the pediatric-onset strategies under discussion (Section 6.5). Adult-onset or acquired hemiparesis populations (e.g., post-stroke) were included only as explicitly flagged cross-population mechanistic analogies, never as direct evidence for pediatric CP.

**Concept:** Rehabilitation strategies, training paradigms, and their underlying neuromuscular, biomechanical, and energetic mechanisms, specifically as they relate to gait asymmetry, motor variability, and gait or postural symmetry as intervention targets or outcomes. Purely diagnostic, classification-, or prevalence-focused studies were included only if they supplied mechanistic or outcome evidence directly usable in Section 3, Section 4, Section 5 and Section 6, not as a general survey of CP epidemiology.

**Context:** Studies published between 2010 and 2026 in English, reporting original research (randomized and non-randomized trials, cohort studies, cross-sectional analyses, and feasibility studies) or systematic reviews/meta-analyses. No restrictions were applied to the rehabilitation setting (clinical, laboratory, or home-based) or country.

### 2.2. Search Strategy

The literature was identified in two search stages using Elicit. An initial stage comprised seven thematically organized search groups; after reviewing this initial evidence base, three supplementary search groups (energy cost and metabolic efficiency; criteria differentiating adaptive from maladaptive asymmetry; and participation/quality-of-life outcomes) were added to address evidentiary gaps identified during synthesis, for a total of ten thematically organized search groups comprising 21 structured search queries. Precise calendar dates for each search stage are available from the authors on request. All queries were executed using Elicit (an AI-assisted literature-discovery tool) and structured as complete research questions rather than keyword strings to maximize retrieval precision. The ten groups addressed were adaptive compensatory asymmetry in hemiplegic CP, pathological symmetric rigidity and motor variability in diplegic CP, robotic and perturbation-based training, gait symmetry as a contested therapeutic goal, neuromuscular control and motor learning, fall risk and balance, long-term musculoskeletal safety, energy cost and metabolic efficiency, criteria differentiating adaptive from maladaptive asymmetry, and participation/quality-of-life outcomes. This search yielded 70 unique sources after de-duplication across groups, comprising the full evidence base retained in Supplementary Table S1 (Section 2.4 addresses the resulting composition of the evidence base); of these 70 sources, all are now cited directly in the reference list of this manuscript: 56 were cited in earlier versions of the manuscript, and the remaining sources retained in the evidence base for background synthesis and evidence-table classification (refs. [7,8,9,10,11,12,13,14,15,16,17,18,19]) have since been added to the main-text reference list and are cited together here, to ensure full alignment between Supplementary Table S1 and the main text bibliography. During peer review, four additional sources (refs. [4,5,6,20]) were incorporated to address specific reviewer comments; these were identified after the search process described above had been completed and are therefore not part of the 70-source evidence base derived from the declared search strategy. They are noted here for transparency, are excluded from Supplementary Table S1 accordingly, and are flagged at their respective points of use in the manuscript (Section 1, Section 3.2 and Section 5.2). The manuscript’s reference list accordingly contains 73 entries in total (56 originally cited from the declared search strategy, plus the 13 additional Table S1 sources noted above [7,8,9,10,11,12,13,14,15,16,17,18,19], plus these 4 peer-review additions). The full query wording and per-group source counts are retained in the accompanying evidence table (Supplementary Table S1) for transparency and reproducibility.

### 2.3. Qualitative Appraisal Methodology

Consistent with the companion review [3], appraisal was informed by general evidence-quality principles associated with the Joanna Briggs Institute (JBI) approach, without applying a formal, design-specific JBI critical appraisal checklist and without a multi-reviewer, dual-independent appraisal process; this review was conducted by a single appraising author, and no formal inter-rater disagreement procedure was therefore required. Instead, each source was evaluated informally for (i) methodological fit to the specific claim it was cited to support (e.g., mechanistic vs. correlational vs. interventional evidence were not treated as interchangeable), (ii) sample characteristics (size, CP subtype, GMFCS level, and spasticity grade) relative to the population defined in Section 2.1, and (iii) whether reported associations were presented by the original authors as correlational or causal, with this distinction preserved rather than collapsed in synthesis (see, in particular, the explicit correlational-not-causal framing maintained throughout Section 3.3). No source was excluded solely based on the study design; single-study or small-sample findings were retained but explicitly flagged as preliminary at the point of use, for example, Section 3.2’s treatment of Elnaggar et al. [21], rather than presented with unwarranted confidence. The instruments underlying several outcome measures cited in Section 3, Section 4, Section 5 and Section 6 have documented psychometric properties supporting their use as intervention outcomes: the Gait Deviation Index and Gait Profile Score show excellent intra-rater reliability in children with spastic CP [22], and the Gait Deviation Index and Gait Variability Index are sensitive to orthosis-induced gait change in hemiplegic CP [23].

### 2.4. Sources of Bias and Mitigation Strategies

Several sources of bias were identified during the review process and are addressed here rather than being left implicit.

**Research group concentration bias.** A substantial portion of the intervention and mechanistic literature on gait-asymmetry magnitude, dynamic balance, and error-augmentation training in unilateral cerebral palsy originates from a small number of active research groups, most notably a single author group responsible for the majority of recent studies on gait-asymmetry indices and their functional correlates [2,21,24,25,26,27], and a second group responsible for the foundational evidence on independent neural control of each limb, drawn from a shared cohort of ten children with hemiplegic CP [28,29]. This concentration is not evidence of bias in either group’s findings, but it constrains the independence of the evidence base: key claims in this review rest on results that have not yet been independently replicated by unrelated research teams or cohorts. Where this concentration bears directly on a specific argument, it is flagged explicitly at the point of use (see Section 3.1 and Section 6.4) rather than treated as a general caveat only.

This concentration extended beyond the hemiplegic arm of the model. The same NIH-affiliated research group responsible for the neural-independence evidence in unilateral CP [28,29] also produced key muscle synergy variability evidence cited for the diplegic/general-CP arm [30,31], meaning a single laboratory contributes mechanistic anchoring to both branches of the trajectory-divergence model rather than to one in isolation. Separately, a single author group [32,33] supplies both the conceptual framing of CP gait as “rigid motor behavior” that motivates the diplegic disruption strategy (Section 4.1) and one of the principal null-transfer findings that qualifies it (Section 4.3)—meaning that the same investigators shape both the theoretical premise and one of its main empirical counterweights. The consequences of this cross-subtype and dual-role concentration for how the model as a whole should be weighted are addressed in Section 6.4.

**Publication and outcome reporting biases.** The literature identified in Group 4 (symmetry as a therapeutic goal) consists almost entirely of studies that frame gait symmetry index improvement as a favorable outcome, with very few sources directly contesting this framing (Section 6, throughout). This asymmetry in the literature itself is treated in this review as substantive evidence of an unexamined disciplinary default (the premise this review contests), rather than as a gap to be apologized for; however, it also means that the “status-quo” characterization applied to several sources in this review, for example, [2,34] reflects an interpretive framing by the present authors, not a claim made by the original authors themselves, who did not set out to test symmetrization as a universal goal.

Database and language coverage: Searches were conducted in English only using a single AI-assisted discovery tool rather than multiple bibliographic databases with independent indexing (e.g., separately run MEDLINE, Embase, and CINAHL). While Elicit draws on large-scale academic indices, its retrieval and ranking behavior have not been independently validated against traditional systematic review search methodology for this specific topic, and non-English literature, which may disproportionately under-represent regions with different rehabilitation traditions or CP-care infrastructure, was not captured.

**Cross-population extrapolation.** The two sources cited in Section 6.5 [35,36] concern adult, acquired hemiparesis (post-stroke) populations rather than pediatric CP. These are explicitly labeled as indirect mechanistic or clinical analogies at each point of use, not as direct evidence for the pediatric CP population defined in Section 2.1, to prevent silent evidentiary substitution.

### 2.5. Analytical Boundaries

This review is a narrative synthesis, not a systematic review or meta-analysis; no formal risk of bias scoring, effect size pooling, or PRISMA-style flow accounting was performed. Its scope is deliberately restricted to hemiplegic and diplegic CP (Section 2.1), to the 2010–2026 publication window, and—per the generalizability constraint established in Section 3.4 and consolidated in Section 6.6—to evidence drawn predominantly from ambulatory children with mild-to-moderate motor impairment (GMFCS I–II). The findings and recommendations should not be extrapolated beyond these boundaries without the qualifications stated at each point in Section 3, Section 4, Section 5, Section 6 and Section 7.

## 3. Conservation of Compensatory Asymmetry in Hemiplegic (Unilateral Spastic) Cerebral Palsy: Mechanisms, Evidence, and Boundaries

### 3.1. Mechanistic Rationale: Asymmetry as the Signature of Independent Limb Control

The gait patterns of children with hemiplegic cerebral palsy (hemi-CP) are persistently asymmetric; however, a growing body of evidence indicates that this asymmetry is not simply the result of an undifferentiated motor deficit. Bulea et al. demonstrated that children with unilateral CP retain separate, independently adaptable neural circuits for each lower limb, capable of adapting kinematics to unilateral leg-weighting without the interference typically expected from a unified, bilaterally coupled control scheme [28]. This finding establishes a capacity for independent limb control. It does not, by itself, demonstrate that conserving asymmetry is therapeutically preferable to correcting it. We treat that further inferential step explicitly as an interpretation developed and qualified across the remainder of this section, rather than as a direct implication of Bulea et al.’s findings. Damiano et al. showed that CP-related motor learning capacity is not categorically impaired relative to typically developing peers. They proposed that error-augmentation training—deliberately exaggerating, rather than minimizing, an existing asymmetry during a training protocol—can enhance motor learning. The authors present this as a training technique for inducing motor adaptation; their study does not test, and should not be read as supporting, the conservation of asymmetry as a rehabilitation endpoint, a distinction we preserve here [29]. Complementary mechanical evidence supports a related interpretation at the level of whole-body coordination: Bruijn et al. found that total-body angular momentum remains tightly regulated in hemiparetic gait despite marked leg asymmetry through compensatory recruitment of the unaffected arm [37]—asymmetry is distributed and controlled, not chaotic.

The “unaffected” limb is an active participant in this compensatory architecture rather than a passive, neutral reference. Huang et al. found that non-affected-limb kinematics and kinetics in spastic hemi-CP are largely a strategy to minimize bilateral stiffness asymmetry across the double-limb-support phase [38], while Tsitlakidis et al. reported that sound-limb kinematics diverge significantly from healthy controls, partly compensating for pelvic malalignment and leg-length discrepancy, without this compensation degrading overall functional outcome [39]. Tabard-Fougère et al. extended this picture kinematically, showing that gait variability is higher on the non-affected side than on the affected side in unilateral CP, consistent with an actively regulating, rather than merely deficient, contralateral system [40].

### 3.2. Functional and Energetic Evidence Dissociating Symmetry from Functional Gain

The central premise of most current hemiplegic gait rehabilitation is that reducing the symmetry index constitutes clinical success (see Section 6 for a systematic account of this paradigm). Preliminary evidence against the necessity of this coupling comes from a single randomized study. Elnaggar et al. found that eight weeks of plyometric training produced significant improvements in walking speed, stride length, and step time in children with unilateral CP without a corresponding significant change in the weight-bearing symmetry index [21]. Because this dissociation currently rests on one trial, it is presented here as suggestive rather than established pending replication.

The energetic literature strengthens this dissociation across a broader range of evidence. Marconi et al. showed that the metabolic cost of walking differs systematically between hemiplegic and diplegic CP subtypes and must be interpreted relative to matched age and speed [41], while Feng et al. quantified an asymmetric division of mechanical work and energy transfer between the affected and unaffected legs in hemiplegic gait [42], which is a compensatory redistribution of effort rather than a generalized inefficiency. Elnaggar and Elfakharany’s analysis of step-length asymmetry drivers further disentangles neuromotor impairment from energy optimization as distinct, partially independent contributors to observed asymmetry [26], offering the closest available empirical basis for separating impairment-driven from efficiency-driven asymmetry—precisely the distinction the trajectory-divergence model requires. Consistent with this, Romkes et al. found that children with unilateral CP retain the capacity to adapt their gait pattern when walking on uneven ground [43], indicating that the compensatory pattern is flexible rather than fixed, which is a further argument against treating it as a rigid deficit requiring correction. A direct, observational test of the asymmetry–energy relationship is available from Gagnat et al. In a retrospective cross-sectional study of 40 ambulant children with CP (GMFCS I-II), they found that gait deviation and height jointly explained approximately 40% of the variance in gross energy cost. Gait asymmetry and muscle co-activation index, however, did not make a significant additional contribution [44]. Two caveats apply to this source specifically. First, Gagnat et al.’s sample was not restricted to hemiplegic CP: it comprised a mixed-subtype CP sample, predominantly GMFCS level I, and its applicability to the hemiplegic arm specifically is therefore inferential in the same way, and requires the same caveat, that we apply to mixed-sample evidence cited for the diplegic arm in Section 4.1. Second, the study’s 71 observations were drawn from 40 children, indicating a repeated-measures structure in which some children contributed more than one observation; this clustering was not addressed in our summary above and should be considered when weighing the independence of the reported association. This finding qualifies, rather than supports, the energetic case made above: it indicates that overall gait deviation, not asymmetry specifically, is the stronger correlate of energy cost in this cross-sectional sample, and we treat it as evidence against, not for, an unconditional asymmetry–energy link.

### 3.3. The Magnitude Boundary: Why Conservation Cannot Be Unconditional

The evidence above supports the treatment of compensatory asymmetry as a legitimate, energy-aware, and adaptable motor strategy rather than a default target for correction. However, two recent studies substantially qualify this position and require the model to be more precise. We use the term “magnitude boundary” throughout this review as a conceptual, hypothesis-level description of a monotonic, dose-like relationship between asymmetry magnitude and functional risk, not as an empirically identified threshold. No study reviewed here defines a validated cut-off, an optimal moderate-range value, or a clinically actionable boundary point, and none should be inferred from the discussion or from Figure 2 below.

Elnaggar [25] examined 85 children with hemi-CP and found that greater temporal and spatial gait-asymmetry indices correlated with poorer dynamic balance control, lower dynamic limits of stability, wider heel-to-heel base of support, lower Dynamic Gait Index score, and slower performance on a timed stair test (weak-to-moderate correlations, r = −0.2 to −0.44) [25]. In a two-year longitudinal study of 27 children with unilateral CP (GMFCS I–II), Elnaggar and Elfakharany found that higher baseline spatial and temporal asymmetry indices were significantly associated with poorer community mobility, functional walking, shuttle-run, and six-minute walk performance, and that children with lower baseline asymmetry improved functionally at a faster rate over 24 months [27]. In both studies, the relationship was monotonic and negative: there was no indication of an optimal non-zero asymmetry magnitude associated with the best functional outcomes; greater asymmetry magnitude was consistently associated with worse, not better, function.

This evidence does not overturn the mechanistic and energetic case made in Section 3.1 and Section 3.2; instead, it constrains it. Both studies are observational; the cross-sectional design of Elnaggar [25] precludes any causal inference, and even the longitudinal design of Elnaggar and Elfakharany [27] establishes association and temporal sequence, not mechanism. Neither study distinguishes why a given child’s asymmetry is large, whether it reflects an efficient, actively regulated compensatory strategy of the kind documented in Section 3.1 and Section 3.2, or a poorly controlled, escalating deviation reflecting deteriorating selective motor control. Therefore, the clinical claim of the trajectory divergence model is not that asymmetry should be preserved unconditionally, but rather that the therapeutic target in hemiplegic CP should be the prevention of asymmetry escalation and the preservation of its adaptive, energy-efficient character, not the forced normalization of the gait pattern toward zero asymmetry. This reframes the hemiplegic arm of the model from a binary contrast with diplegia (“preserve vs. disrupt”) into a magnitude- and quality-sensitive target, which is both more defensible against the correlational evidence above and more clinically actionable, since it converts an abstract preservation goal into a monitorable boundary condition (addressed operationally through the DRRF framework in Section 5).

Figure 2 depicts this magnitude boundary graphically: the risk to balance and locomotor capacity rises monotonically with asymmetry magnitude (consistent with the correlational evidence above), with no support for the alternative view that reducing asymmetry toward zero improves outcome. The therapeutic implication is therefore directional—prevent further rightward drift—rather than a fixed target of symmetrization.

### 3.4. Generalizability Constraint

The evidence base underlying Section 3.1, Section 3.2 and Section 3.3 is drawn predominantly from ambulatory children with mild-to-moderate hemi-CP: GMFCS levels I–II across nearly all cited studies, and, where spasticity grading is reported [25], restricted to Modified Ashworth Scale 1/1+. Therefore, the mechanistic and magnitude-boundary arguments developed here should be read as applying to this ambulatory, mild-to-moderate severity band; extension to children with more severe spasticity, lower GMFCS levels, or non-ambulatory presentations is not supported by the current evidence and is flagged as an open question for the research agenda in Section 7.

### 3.5. Synthesis

Taken together, the mechanistic (Section 3.1), functional/energetic (Section 3.2), and magnitude-boundary (Section 3.3) evidence bases converge on a single, qualified proposition: hemiplegic gait asymmetry is, at moderate magnitudes and within the ambulatory, mild-to-moderate severity range described in Section 3.4, an actively regulated, energy-aware, independently controlled compensatory strategy that need not—and does not always need to—be corrected to achieve functional gain; however, its magnitude is not therapeutically inert, and unchecked escalation is associated with deteriorating balance and locomotor capacity. This distinguishes the position argued here from both poles of current practice: the correction-oriented paradigm that treats any residual asymmetry as a deficit (critiqued in Section 6), and an uncritical “asymmetry is always adaptive” stance that the data in Section 3.3 do not support.

**Bridge to Section 6.** Section 6 examines a set of hemiplegic CP intervention studies, most notably split-belt treadmill protocols using error augmentation [24,45], in which actively reducing gait asymmetry produced measurable functional and balance gains in the same population addressed here. These findings are not treated as contradicting the magnitude-boundary framework proposed in Section 3.3; rather, Section 6 reconciles them by considering whether the asymmetry reduced in those protocols reflects the escalating maladaptive component the present model targets for prevention, rather than the moderate compensatory asymmetry defended in Section 3.1 and Section 3.2.

## 4. Disruption of Pathological Rigid Symmetry in Diplegic (Bilateral Spastic) Cerebral Palsy: Mechanisms, Evidence, and Boundaries

### 4.1. Mechanistic Rationale: Reduced Variability as the Signature of Motor-Control Rigidity

Where hemiplegic gait is characterized by a persistent, actively regulated asymmetry (Section 3), bilateral spastic (diplegic) cerebral palsy presents a contrasting signature: a loss of the adaptive variability that characterizes typical motor control, expressed as an excessively rigid, over-coupled coordination pattern, rather than a controlled asymmetric compensation. Dussault-Picard et al. found that children with cerebral palsy show more in-phase knee–hip and ankle–knee coordination than typically developing peers, together with lower coordination variability at several points in the gait cycle—direct kinematic evidence of reduced inter-joint adaptability [46]. At the neuromuscular level, this rigidity is paralleled by a reduction in motor control complexity. Bekius et al.’s systematic review found that children with CP recruit fewer muscle synergies during walking than typically developing children, with synergy characteristics that can predict treatment outcomes [47]. Hashiguchi et al. confirmed that the number of synergies decreases with increasing spasticity severity [48]. The evidence above should be read with two qualifications. First, none of these three studies were restricted to bilateral spastic (diplegic) samples: Dussault-Picard et al., Bekius et al., and Hashiguchi et al. studied heterogeneous, non-subtype-stratified CP samples, and further evidence discussed below (Bar-Haim et al. [32]) combines participants with CP and post-stroke hemiparesis. The applicability of this mechanistic case specifically to diplegic CP is therefore an inference from broader CP and mixed-population evidence, not a direct demonstration in bilateral spastic samples, and is flagged as a limitation in Section 6. Second, “rigidity” and “adaptive variability” are used here to denote distinct constructs measured at different levels, which should not be treated as interchangeable: inter-joint coordination variability (Dussault-Picard et al.), muscle synergy count (Bekius et al.; Hashiguchi et al.), and muscle synergy activation variability (Kim et al., discussed below) are separate measures, and a reduction in one does not imply a reduction in another.

Bar-Haim et al. explicitly state this framing, characterizing CP and hemiparetic gait as “rigid motor behavior that prevents adaptation to changing environmental conditions” [32]—a formulation that positions rigidity, not asymmetry, as the pathological target in the diplegic arm of the trajectory-divergence model. Kim et al. add an important nuance to this picture: although kinematic coordination becomes more rigid, muscle synergy activation shows greater stride-to-stride variability in CP than in typically developing children [30], and CP-specific synergies are more sensitive to environmental perturbation than typically developing synergies [31]. Rigidity in diplegic gait therefore does not describe a uniformly reduced variability across all levels of motor organization, but specifically a loss of adaptive, task-appropriate variability at the coordination level—a distinction that motivates perturbation- and variability-based intervention (Section 4.2) rather than variability-suppressing approaches.

### 4.2. Evidence for Variability-Restoring Interventions

Direct support for a disruption strategy comes from Kwon and Ahn, who found that high-variability practice combined with task-oriented training produced greater improvement in gross motor performance and activities of daily living independence in children with spastic diplegia than neurodevelopmental treatment alone [49]. Mechanistically, this is consistent with the broader perturbation-training literature: Tang et al. showed that gradual, rather than abrupt, perturbation loading produces longer retention of locomotor adaptation in children with CP [50], and Yan et al. demonstrated that continuous lateral trunk-support perturbation improves trunk postural control, which subsequently transfers to improved overground walking speed [51]. Using an explicitly chaotic perturbation paradigm, Bar-Haim et al. found that 12 weeks of training with a mechatronic perturbation device shifted complexity indices in individuals with CP and hemiparesis toward values seen in unimpaired controls [32]. Phillips et al. extended this to a robot-assisted, side-specific compliant-surface protocol, reporting improved functional balance and reduced fall risk (via increased time-to-contact), an important reassurance for the safety domain of a disruption strategy [52].

Robotic gait training offers an additional avenue for inducing variability under controlled conditions. Žarković et al. reported that four weeks of robot-assisted gait training changed muscle-activation timing and hip range of motion in children with diparesis [53], and gains in gait training have been more broadly linked to improved push-off force alongside gait symmetry in children with CP [54].

### 4.3. Boundaries: Subtype Heterogeneity and Mixed Efficacy Evidence

As with the hemiplegic arm of the model (Section 3.3), the disruption strategy for diplegia cannot be stated unconditionally for two distinct reasons.

First, the underlying premise that diplegia entails uniform bilateral symmetry does not hold for a substantial minority of children with diplegia. A single conference abstract report by Gahukamble, based on a video analysis of 50 ambulatory children with bilateral spastic CP, reported that approximately 32% showed a clinically meaningful difference in gait deviation severity between the two sides (Edinburgh Visual Gait Score difference ≥4) [55]. As a conference abstract, this report has not undergone full peer review, provides limited methodological detail, and has not, to our knowledge, been independently replicated. This figure should therefore be read as a preliminary signal rather than an established prevalence estimate. This directly complicates a clean hemiplegic-asymmetric versus diplegic-symmetric dichotomy: a meaningful subgroup of children classified as diplegic presents with their own inter-limb asymmetry, for which the hemiplegic arm’s magnitude-boundary logic (Section 3.3), rather than the diplegic disruption strategy, may be more directly applicable. If confirmed by future, fully peer-reviewed research, this heterogeneity would represent a meaningful qualification of the model rather than a peripheral exception; it is addressed further, with this evidentiary caveat, as a model limitation in Section 6.

Second, the efficacy evidence for variability- and perturbation-based interventions is considerably more mixed than the mechanistic rationale in Section 4.1 and Section 4.2 suggests. Manikowska et al. found that robot-assisted therapy produced no significant change in spatiotemporal parameters or the Gait Deviation Index (GDI) in children with bilateral spastic CP, with only limited improvement in double support and walking speed symmetry [56]. We note that, unlike the Gait Profile Score, for which a minimal clinically important difference (MCID) of approximately 1.6° has been established, no validated MCID has, to our knowledge, been established specifically for the GDI. It therefore cannot currently be determined whether a statistically significant GDI change in this population would also have been clinically meaningful. We can, however, situate the result against measurement precision. Rasmussen et al., already cited in this review [22], reported a smallest detectable change (SDC) for the GDI of approximately 11.3–18.5%. This means that Manikowska et al.’s non-significant finding is also consistent with a true change that simply fails to exceed the instrument’s own measurement error—independent of the separate question of clinical meaningfulness. Van Kammen et al. found that Lokomat guidance and body-weight support reduce active muscle-activation amplitude relative to free treadmill walking [57], raising the concern that heavily assisted robotic protocols may work against the variability-restoring goal they are intended to serve. In young adults with CP, Bar-Haim et al. found that repeated split-belt training improved adaptation rate, reduced step-by-step variability, and improved center-of-pressure symmetry, with retention at six months, but produced no significant transfer to functional, everyday overground walking ability [33]; we note that [33] is a one-page conference abstract rather than a full peer-reviewed article, which limits the methodological detail available and should temper the evidentiary weight placed on this null-transfer finding. Most directly, Afzal et al. compared perturbation-based training with trunk-control training in children with CP and found that trunk-control training produced significantly greater improvement across all outcome measures assessed: the Trunk Control Measurement Scale, Pediatric Balance Scale, Lower Extremity Functional Scale, and radiographic spinal alignment [58]. This is direct counter-evidence that perturbation/variability-based approaches are not uniformly superior to more conventional postural-control training and is addressed as a complicating case in Section 6, in parallel with the split-belt hemiplegic studies flagged in Section 3.5.

### 4.4. Synthesis

The mechanistic (Section 4.1) and interventional (Section 4.2) evidence supports a disruption-oriented rehabilitation target for diplegic CP: restoring adaptive, task-appropriate motor variability at the level of inter-joint coordination and, where feasible, muscle synergy activation, rather than pursuing further gait “correction” toward an already rigid symmetric pattern. However, this target should be applied conditionally on two grounds established in Section 4.3: it presupposes that the child’s presentation reflects genuine bilateral rigidity rather than an unrecognized inter-limb asymmetry (present in roughly a third of ambulatory bilateral spastic CP cases), and the strength of transfer from laboratory-demonstrated variability gains to durable, functional, everyday improvement remains inconsistent across the current intervention literature, ranging from encouraging [49,52] to null or reversed [33,58].

Table 1 summarizes the two arms of the model side by side, drawing together the mechanism, therapeutic target, evidentiary strength, and boundary condition established in Section 3 and Section 4.

**Bridge to Section 6.** Section 6 revisits the findings of Manikowska [56], Bar-Haim [33], van Kammen [57], and Afzal [58] together with the hemiplegic split-belt studies (Section 3.5) as a joint discussion of when symmetry- or rigidity-altering interventions fail to produce durable functional transfer across both CP subtypes and considers whether this reflects a shared methodological limitation (dosage, assistance level, or outcome selection) rather than a failure of the trajectory-divergence model itself.

## 5. The Divergent Rehabilitation Response Framework (DRRF): A Provisional Heuristic for Congruence-Based Monitoring

### 5.1. Status and Purpose

The Divergent Rehabilitation Response Framework (DRRF), introduced provisionally in the model-building stage of this review, is a heuristic construct and not a validated clinimetric instrument. It has no scoring algorithm, no established psychometric properties, and no claim of measurement equivalence with existing gait symmetry indices. Its function here is narrower and more specific: to organize, domain by domain, what the evidence assembled in Section 3 and Section 4 implies about congruence—the degree to which a child’s response to a given rehabilitation strategy matches the direction predicted by their subtype-specific trajectory (asymmetry-bounded for hemiplegia, rigidity-disrupting for diplegia)—rather than measuring gait symmetry directly, as conventional indices do. Section 3 and Section 4 asked whether the mechanistic and interventional evidence supports each subtype’s target, while Section 5 asked a narrower, more operational question: what would count as evidence that the target is being met, and how far does the current literature already answer that question for each of the four proposed domains?

### 5.2. Domain-by-Domain Evidentiary Status

**D1—Compensatory/variability efficiency (CV).** For hemiplegia, this domain asks whether asymmetric compensation is metabolically efficient rather than being costly. The evidence is directionally supportive but incomplete: Marconi et al. established that energy cost differs systematically by subtype and must be interpreted at matched age/speed [41]; Feng et al. quantified an asymmetric but coordinated division of mechanical work between limbs, consistent with efficient compensation rather than generalized inefficiency [42]; and Elnaggar and Elfakharany’s analysis of step-length asymmetry drivers is the only source to date that statistically separates energy optimization from neuromotor impairment contributions to asymmetry [26]. One observational study has directly tested the asymmetry–energy relationship: Gagnat et al. found that gait asymmetry did not significantly contribute to energy cost once overall gait deviation was accounted for, in a cross-sectional sample of ambulant children with CP [20]; however, no interventional study has tested whether an intervention preserving asymmetry produces a lower energy cost than one correcting it, and the single RCT bearing on functional gain without symmetrization [21] did not measure energy cost. Therefore, D1 for hemiplegia is only partly and inconsistently supported by direct evidence, rather than simply untested; the available cross-sectional evidence does not confirm that asymmetry itself is the energetically relevant feature.

For diplegia, D1 concerns the restoration of adaptive variability rather than efficiency. Kwon and Ahn provide the clearest interventional evidence: high-variability practice improved gross motor performance and ADL independence more than standard treatment [49]. Dussault-Picard et al. establish the baseline deficit this targets—reduced inter-joint coordination variability relative to typically developing peers [46]. D1 for diplegia is better supported by direct intervention evidence than its hemiplegic counterpart, although still from a single trial.

**D2—Independent neural control/adaptive response.** For hemiplegia, this domain is the most directly evidenced of the four, resting on the Bulea/Damiano cohort’s demonstration of preserved, independently adaptable neural circuits per limb [28,29]—subject to the evidentiary-concentration caveat already flagged in Section 2.4 and Section 6.4. For diplegia, D2 concerns the emergence of adaptive, functional response to perturbation as a marker of restored control: Yan et al.’s studies show that perturbation-based training with varied, rather than constant, force magnitude more effectively improves trunk postural control, and that baseline motor variability predicts the degree of subsequent motor learning [51,59,60]. This is directly relevant, since it suggests that D2 in diplegia may be self-limiting: children with less baseline variability may show a weaker congruence signal regardless of intervention quality. This is a nuance not previously captured in the model.

**D3—Participatory functionality.** This domain was the least evidenced when the DRRF was first proposed and has since been substantially strengthened for hemiplegia. Elnaggar and Elfakharany’s longitudinal study directly measured functional/participation-adjacent outcomes (community mobility, functional walking, and six-minute-walk distance) against asymmetry magnitude over 24 months [27]; Çelik and Kemer examined gait- and balance-related factors affecting school-aged participation directly [61]; Coello-Villalón et al.’s systematic review and meta-analysis is the best available anchor for whether specific categories of rehabilitation intervention differentially improve participation outcomes in unilateral CP [62]; and Eltahan et al. found a direct relationship between postural asymmetry and quality of life in children with hemiparesis [63], further reinforcing the participation-adjacent relevance of this domain. Critically, the magnitude-boundary finding from Section 3.3 directly qualifies D3: because higher asymmetry indices correlate with worse, not better, functional capacity trajectories in the same longitudinal cohort [27], D3 cannot be scored simply as “preserved asymmetry = better participation”; it must be read jointly with D1 and the magnitude constraint, since the participation gain the model predicts is conditional on asymmetry remaining within the moderate, non-escalating range described in Section 3.3.

For diplegia, D3 is comparatively under-evidenced. Kwon and Ahn’s ADL-independence finding is the only direct participation-level evidence identified [49]; the null/mixed functional-transfer findings from Section 4.3 [33,56,58] bear more directly on D3 than on D1/D2, since they specifically concern whether laboratory-level variability or balance gains translate into durable, real-world functional improvement. Therefore, D3 for diplegia is the domain with the weakest current support, not because the construct is implausible, but because the intervention literature that would test it most directly returns inconsistent results.

**D4—Risk/safety.** For hemiplegia, the evidence is reassuring on balance: Tracy et al. found that children with CP adopt a more conservative, protective lateral-stability strategy when weight-bearing on the non-dominant limb, consistent with asymmetric compensation serving a protective rather than destabilizing function [64]; Boyer and Patterson found no association between specific gait-pathology subtypes and self-reported fall frequency [65]; and Bentley et al. found fall frequency associated with strength-/balance-test performance rather than gait pattern per se [66]. However, this is set against the magnitude-dependent finding of Elnaggar [25], which states that greater asymmetry correlates with lower dynamic limits of stability [25]. D4 for hemiplegia is therefore best read as domain-general reassurance (asymmetry as such does not appear to elevate fall risk) combined with a magnitude-specific caution (very large asymmetry does correlate with reduced dynamic balance)—the same qualification structure as D1/D3.

For diplegia, D4 evidence is directly relevant to the safety of a disruption strategy: Phillips et al. found that perturbation-based training on compliant surfaces reduced fall risk (increased time-to-contact) while improving dynamic postural balance [52]—reassuring. Afzal et al.’s finding that trunk-control training outperformed perturbation training on spinal alignment and balance outcomes [58] complicates this domain in the same way it complicates D3: perturbation-based approaches are not shown to be uniformly safer or more effective than conventional postural training, and D4 should not be read as unconditionally favoring the disruption strategy over alternatives.

Table 2 consolidates the evidentiary status of all four domains for both subtypes, summarizing the discussion above in a single reference view.

### 5.3. Boundaries of the DRRF as Currently Constituted

Three limitations apply to the DRRF, as presented here, and should be stated plainly rather than implied. First, no domain has a proposed measurement protocol, scoring rules, or thresholds. The discussion above reports the direction and strength of the available evidence per domain, not a scale. Second, the domains are not evidentially independent of one another: the magnitude-boundary constraint identified in Section 3.3 propagates into D1, D3, and D4 for hemiplegia simultaneously, since all three are conditional on asymmetry remaining within a moderate, non-escalating range rather than being a free-standing “more asymmetry is better” claim. Third, and most importantly, the DRRF has not been prospectively applied to any cohort; every citation supporting a given domain was identified retrospectively from studies not designed to test the DRRF construct. Therefore, the index should be read as a synthesis scaffold for interpreting the existing literature through a congruence lens and as a proposed direction for future prospective operationalization (Section 7), not as a ready-to-use clinical or research tool.

### 5.4. Synthesis

Across both subtypes, the DRRF domains were unevenly distributed. D2 (neural independence/adaptive response) is the best-anchored domain for hemiplegia, although narrowly so (Section 2.4/Section 6.4 concentration notes); D1 (variability restoration) is the best-anchored domain for diplegia, although resting on a single trial. D3 (participation) has strengthened considerably for hemiplegia following the three supplementary search groups added in the second search stage (Section 2.2) but remains the weakest domain for diplegia. D4 (safety) is reassuring in direction for both subtypes but is, in both cases, qualified by evidence that the relevant intervention or asymmetry magnitude is not uniformly protective. No domain, for either subtype, currently supports an unconditional reading; every domain inherits, in some form, the magnitude- or transfer-related qualifications established in Section 3.3 and Section 4.3 below.

**Bridge to Section 6.** Section 6 draws together the domain-level qualifications noted here—the magnitude boundary (Section 3.3), the subtype-heterogeneity boundary (Section 4.3), the mixed transfer evidence (Section 4.3), and the evidentiary-concentration notes (Section 2.4)—into a single, integrated account of the model’s limitations, before turning to the long-term musculoskeletal safety literature not yet incorporated into the DRRF’s D4 domain.

## 6. Contra-Arguments and Limits

### 6.1. Reconciling Correction-Beneficial Cases with the Trajectory-Divergence Framework

Section 3.5 and Section 4.4 both flagged intervention studies in which reducing, rather than preserving, subtype characteristic gait patterns produced measurable functional gains. These cases require direct reconciliation rather than selective omission.

Hemiplegia. Elnaggar et al. found that 12 weeks of split-belt treadmill training using an error-augmentation strategy—deliberately exaggerating the child’s initial step-length asymmetry—produced significantly more favorable step-length asymmetry reduction, improved limits of stability, and greater locomotor capacity than conventional single-belt training in adolescents with unilateral CP [24]. The published protocol confirms that this exaggeration of the child’s initial step-length asymmetry was the training manipulation, consistent with an error-augmentation, rather than error-reduction, design. However, the exact belt-speed ratio and which limb was assigned to the faster belt are not stated in the available abstract-level description; this detail should be confirmed against the full published protocol before the error-augmentation classification is treated as definitive. Sorek et al. similarly found, in a one-page conference abstract [45], that a single session of split-belt walking shifted bilateral muscle synergy activation patterns toward a typically developing structure and improved step-length symmetry; as with [33], the conference abstract format limits the methodological detail available and warrants cautious interpretation. A further correction-beneficial case comes from El-Shamy and Abdelaal, who found that functional electrical stimulation (WalkAide) aimed at correcting the hemiplegic gait pattern also reduced energy expenditure [67], a case in which a correction-oriented intervention improved, rather than worsened, metabolic efficiency. At face value, these findings appear to contradict the conservation argument presented in Section 3.

We propose the following reconciliation, offered as an interpretation consistent with the evidence rather than as an established finding: all three protocols used active, principle-driven intervention—error augmentation grounded in the independent-neural-control mechanism defended in Section 3.1 [29] or targeted neuromuscular stimulation—as the means of inducing change, not a direct corrective force applied uniformly to the resting gait pattern. The resulting reduction in resting asymmetry or energy cost is therefore plausibly consistent with the magnitude-boundary framework of Section 3.3: these training protocols may reduce an elevated, escalating, or maladaptive component of asymmetry toward a moderate, functionally compensatory range, rather than eliminating compensatory asymmetry altogether. Critically, none of the three studies reported where post-treatment asymmetry values fall relative to a “zero symmetry” target versus a moderate compensatory range; therefore, this reconciliation cannot currently be confirmed against the data and should be treated as a hypothesis for future work (Section 7), not a resolved tension.

**Diplegia.** Afzal et al. found that trunk-control training outperformed perturbation-based training across all measured outcomes in children with CP [58], complicating the disruption strategy defended in Section 4.1 and Section 4.2. Unlike the hemiplegic cases above, this is not straightforwardly reconcilable with the trajectory divergence model as currently specified because trunk-control training is not a variability-restoring intervention in the sense argued for in Section 4.1; it is closer to conventional postural strengthening. A plausible but speculative explanation is a sequencing effect, in which a minimum threshold of postural control may be a prerequisite for perturbation-based variability training to be effective, such that perturbation training delivered without adequate baseline trunk control underperforms targeted strengthening first. This explanation has not been tested in any study identified in this review and is offered here only as a candidate hypothesis, not a resolution.

### 6.2. Subtype Heterogeneity as a Structural Limitation of the Model

The trajectory-divergence model’s binary framing—persistent asymmetry in hemiplegia versus pathological rigid symmetry in diplegia—does not hold cleanly for a meaningful subset of children. A single, not yet independently replicated conference abstract (Gahukamble [55]), based on a video-based study of 50 ambulatory children with bilateral spastic CP, reported that approximately 32% showed clinically meaningful interlimb asymmetry in gait deviation severity; given its conference abstract status and limited methodological reporting, this figure should be treated as a preliminary signal, not an established prevalence estimate. For this subgroup, the hemiplegic arm’s magnitude-boundary logic (Section 3.3) and not the diplegic disruption strategy may be the more clinically appropriate framework despite a bilateral diagnosis. This is a genuine structural tension in a subtype-based framework, not a minor exception. We considered reformulating the model as fully phenotype-based (defined by measured asymmetry-and-variability profile rather than clinical diagnosis) rather than subtype-based (defined by hemiplegic/diplegic diagnosis). We retain the subtype-based framing for three reasons, while acknowledging its limits. First, clinical diagnosis (hemiplegic vs. diplegic) is what is actually available to clinicians at the point of rehabilitation planning, before any detailed instrumented asymmetry-variability profiling has been performed; a purely phenotype-based model, however conceptually cleaner, would not be actionable without the kind of routine instrumented screening we call for as a research priority in Section 7.2. Second, the mechanistic evidence in Section 3 and Section 4 was itself generated and reported by subtype, not by measured phenotype, so a fully phenotype-based reformulation would currently outrun the evidence base it would need to rest on. Third, we view the approximately one-third heterogeneity rate, if confirmed, as evidence that subtype should be treated as a strong prior to be checked by phenotypic assessment, not discarded outright; this is precisely the intent of the DRRF’s congruence framing (Section 5) and of Recommendation 5 (Section 7.1). We therefore present phenotype-based reclassification as a natural direction for future validation and instrument development, and we revise the recommendations in Section 7.1 to specify inter-limb asymmetry assessment as a precondition for subtype-based strategy selection, rather than as a wholesale replacement for the subtype framework at this stage of the evidence base.

### 6.3. Mixed and Null Functional-Transfer Evidence

Beyond the reconciliation attempted in Section 6.1, several findings indicate that laboratory-demonstrated gains in symmetry, balance, or variability do not consistently translate into durable, real-world functional improvement, independent of which subtype or strategy is considered. Manikowska et al. found no significant change in spatiotemporal parameters or the GDI following robot-assisted therapy in bilateral spastic CP, with only limited symmetry improvement [52]; as noted in Section 4.3, no validated MCID exists for the GDI, and the smallest detectable change reported for the GDI (approximately 11.3–18.5%; Rasmussen et al. [22]) indicates that this null result is also consistent with a change too small to exceed measurement error, so the clinical meaningfulness of this result, had it instead reached statistical significance, cannot be independently assessed. Van Kammen et al. found that Lokomat guidance and body-weight support reduce active muscle contribution relative to free treadmill walking [57], raising concerns that heavily assisted protocols may work against their own variability-restoring intent. Bar-Haim et al. found that repeated split-belt training in young adults with CP improved adaptation rate, step-variability reduction, and center-of-pressure symmetry with six-month retention but produced no significant transfer to functional overground walking [33] (a conference abstract source; see the caveat in Section 6.1).

Taken together with Afzal’s [58] finding in 6.1, this pattern is not specific to one subtype or one intervention family; it recurs across robotic, split-belt, and perturbation-based protocols in both hemiplegic and diplegic samples. We treat this as evidence of a shared methodological limitation across the current intervention literature, likely involving dosage, level of assistance, and choice of outcome measure (impairment vs. functional/participation-level), rather than as evidence against the trajectory-divergence model itself. This interpretation is offered as the most parsimonious reading of a heterogeneous evidence base, not as a settled conclusion, and is proposed as a specific target for future research design in Section 7 of this paper.

### 6.4. Evidentiary Concentration and Dual-Role Research Groups

The mechanistic pillar of Section 3.1 rests on a single ten-child cohort reported by one research group [28,29], which has not yet been independently replicated. This concentration extends across, not just within, subtypes: the same laboratory contributes muscle synergy variability evidence to the diplegic/general-CP arm [30,31], meaning a methodological artifact specific to that group’s cohort or analytic pipeline could, in principle, affect both branches of the model simultaneously. Separately, Bar-Haim et al. supply both the conceptual premise motivating the diplegic disruption strategy (Section 4.1) and one of the clearest null-transfer results qualifying it (Section 4.3 and Section 6.3), meaning that part of the apparent internal tension in Section 4 reflects a tension within a single research program rather than disagreement between independent investigators. Readers should weigh the model’s mechanistic claims accordingly, pending broader replication.

### 6.5. Long-Term Musculoskeletal Safety of Conserved Compensatory Asymmetry

The conservation argument in Section 3 requires confrontation with the strongest available counterargument: that chronic compensatory asymmetry may carry a long-term musculoskeletal cost not visible in the pediatric outcome measures used throughout Section 3, Section 4 and Section 5. The epidemiological evidence is consistent and concerning. French et al. found significantly elevated adjusted odds of any, poly-, hip, knee, and other/unspecified osteoarthritis in adults with CP compared with matched controls (OR range 1.3–2.1, *p* < 0.001 for each site), but found no significant difference for hand osteoarthritis specifically (OR 0.86, *p* = 0.46); confidence intervals for individual joint-level estimates were not reported in the source publication’s abstract and should be obtained from the full text where a site-specific estimate is required [68]. In a population-based cohort, O’Connell et al. found an increased adjusted risk of osteoporosis (HR 6.19, 95% CI 3.37–11.39, *p* < 0.001) and, after adjusting specifically for alcohol consumption, smoking status, and mean yearly general-practice visits, osteoarthritis (HR 1.54, 95% CI 1.17–2.02, *p* < 0.001) [69]. The authors note that residual confounding by mobility limitation, body mass, or joint-loading pattern cannot be excluded. Attributing this risk specifically to gait asymmetry, as opposed to CP-associated immobility or other factors more broadly, would therefore not be justified. Ryan et al.’s 2026 systematic review confirmed a higher burden of osteoarthritis, osteoporosis, and fracture in adults with CP, albeit with low-to-moderate certainty evidence [70].

Critically for the conservation argument specifically, Tretiakov et al. found that the “unaffected” hip in hemiplegic CP is not a neutral reference: its static internal rotation correlates strongly with that of the affected hip, and when affected-hip rotation is severe, the unaffected side’s parameters are also abnormal [44]. Cross-population mechanistic evidence from post-stroke gait—cited here as an indirect analogy, not direct CP evidence—reinforces the plausibility of this concern: Giarmatzis et al.’s musculoskeletal modeling found consistently higher medial compartment knee loading in the non-paretic limb than in the paretic limb [36], a mechanical pathway by which conserved compensatory loading on the “good” side could predispose the joint to degeneration over decades.

Hemiplegia-specific long-term trajectory data offer a more direct comparison than general adult CP epidemiological literature. Schranz et al.’s long-term follow-up of unilateral single-event multilevel surgery in spastic hemiplegia [71] and Bonnefoy-Mazure et al.’s 10.5-year follow-up of gait evolution in unilateral spastic CP [72] together provide the closest available long-term, subtype-specific comparators, although neither directly tests the conservation strategy proposed in Section 3.

This evidence does not, on its own, refute the conservation argument; none of the cited epidemiological studies isolated compensatory gait asymmetry as the causal mechanism for elevated musculoskeletal risk in adults with CP, as opposed to reduced mobility, altered bone density, spasticity-related joint loading, or other CP-associated factors. However, this means that the conservation strategy proposed in Section 3 cannot be evaluated on pediatric functional outcomes alone; adult, long-term musculoskeletal evidence must also be weighed. A countervailing data point offers some reassurance: Lennon et al.’s cohort, followed from adolescence into adulthood under specialized orthopedic care, showed no clinically meaningful decline in the Gait Deviation Index or gross motor function [73], suggesting that long-term outcomes may depend substantially on monitoring and care quality, not on asymmetry conservation per se. The honest position is that the long-term safety of a magnitude-bounded conservation strategy (Section 3.3) is currently unknown and should be treated as an open empirical question, not resolved in either direction by the available literature—a priority explicitly carried forward into the research agenda (Section 7).

### 6.6. Generalizability Constraints

The evidence base underlying Section 3, Section 4 and Section 5 skews toward ambulatory children with mild-to-moderate impairment: GMFCS levels I–II predominate throughout, and where spasticity grading is reported [25], the sample is restricted to Modified Ashworth Scale 1/1+. The model’s claims—for both the hemiplegic magnitude boundary (Section 3.3) and the diplegic disruption strategy (Section 4)—should be read as applying to this ambulatory, mild-to-moderate severity band. The extension of this study to children with more severe spasticity, lower GMFCS levels (III–V), or non-ambulatory presentations is not supported by the current evidence. A related limitation concerns sample composition rather than severity: the mechanistic evidence for diplegia-specific rigidity (Section 4.1) draws substantially on studies of heterogeneous, non-subtype-stratified CP samples (Dussault-Picard et al., Bekius et al., Hashiguchi et al.) and, for the conceptual framing of gait as “rigid motor behavior,” on a sample combining CP and post-stroke hemiparesis (Bar-Haim et al. [32]). The diplegia-specific applicability of this mechanistic case is therefore inferred from broader and mixed-population evidence rather than directly demonstrated in bilateral spastic samples and should be read with this qualification in mind alongside the severity restriction above.

### 6.7. Synthesis

None of the limitations identified in this section overturns the trajectory divergence model or the divergent rehabilitation target argument built on it in Section 3, Section 4 and Section 5. Taken together, however, they establish that the model’s current form is subtype-conditional rather than diagnosis-conditional (Section 6.2); dependent on training principle rather than resting-pattern correction, in ways not yet fully verified against outcome data (Section 6.1); built on an evidence base with real, partially overlapping concentration across research groups (Section 6.4); silent on long-term musculoskeletal consequences, which existing epidemiological evidence renders a live concern rather than a remote one (Section 6.5); and bounded to a specific ambulatory, mild-to-moderate severity population (Section 6.6). These are presented as the honest boundaries of a genuinely novel argument, not as concessions that weaken the argument. A review that contests the prevailing symmetrization paradigm (Section 4 and Section 6.1) without thoroughly confronting its own evidentiary limits would be less credible and not more persuasive.

## 7. Differentiated Clinical Recommendations and Research Agenda

### 7.1. Clinical Recommendations

The recommendations below are stated as conditional, not categorical, in direct response to the boundaries established in Section 6. None of these should be read as a standalone protocol; each presupposes the preconditions and monitoring caveats attached to it. These recommendations are not graded using a formal system such as GRADE, and we did not conduct the systematic, structured evidence synthesis that such grading requires; however, to avoid conveying uniform confidence, we characterize the certainty underlying each recommendation qualitatively: Recommendation 2 rests on multiple converging correlational sources and is considered moderate-certainty. Recommendations 1, 3, 6, and 7 are considered low-certainty: Recommendation 1 rests on a single RCT dissociating functional gain from symmetrization (Section 3.2) together with an observational energy-cost study (Gagnat et al.) that does not unambiguously support the underlying energetic mechanism; Recommendation 3 rests on a single RCT-level reconciliation hypothesis; Recommendation 6 rests on direct interventional evidence from a single small trial (Table 2) together with inconsistent functional transfer noted in Section 4.4; and Recommendation 7 rests on a single RCT (Afzal et al.). Recommendation 5 rests substantially on a single conference abstract prevalence estimate (Section 6.2) and is very-low-certainty pending replication. Recommendations 4 and 8 are boundary-setting or precautionary in nature rather than directly evidence-graded. These certainty levels are also reported directly in Table 3, alongside each recommendation. Readers should weight each recommendation accordingly rather than treating the imperative phrasing below as uniform across the set.

Table 3 reformats these eight recommendations for quick clinical reference, cross-linked to the section of the review that grounds each recommendation.

**For hemiplegic CP:**

1. Residual gait asymmetry should not be treated as a default correction target. If a child’s compensatory asymmetry falls within the moderate range associated with preserved function in Section 3.1 and Section 3.2, rehabilitation goals should prioritize functional gain (speed, efficiency, and participation) over symmetry index reduction as a primary outcome. This is a provisional, hypothesis-driven recommendation based on short-term functional evidence (Section 3.1 and Section 3.2); it should not be read as a directive to deliberately maintain or prolong asymmetric loading, since, as discussed in Section 6.5, the long-term musculoskeletal safety of conserved compensatory asymmetry is currently unknown. Routine long-term monitoring (Recommendation 8) should accompany this approach pending prospective safety evidence.

2. Monitor the asymmetry magnitude, not merely its presence. Given the magnitude-dependent findings in Section 3.3, periodic reassessment of asymmetry indices alongside dynamic balance and functional capacity measures is warranted to detect escalation, which the evidence associates with deteriorating, not improving, function.

3. Where correction-oriented protocols are used (e.g., error-augmentation split-belt training and targeted FES), outcomes should be evaluated against a moderate asymmetry target, not a zero-symmetry target. This directly operationalizes the reconciliation hypothesis proposed in Section 6.1 and would, if adopted, generate the data needed to test it.

4. Restrict application to the evidenced population (ambulatory, GMFCS I–II, mild-to-moderate spasticity) pending evidence in more severely affected or non-ambulatory children (Section 6.6).

**For diplegic CP:**

5. Confirm bilateral symmetry before applying a disruption-based strategy to the network. Given that approximately one-third of ambulatory children with bilateral spastic CP present with clinically meaningful inter-limb asymmetry (Section 6.2), clinical or instrumented asymmetry screening should precede strategy selection; children with diagnosed diplegia but confirmed inter-limb asymmetry may be better served by the hemiplegic magnitude-boundary approach (Recommendations 1–3) than by variability-restoring interventions.

6. Favor variability-restoring approaches (task-oriented high-variability practice, graded perturbation training) over further static correction, consistent with Section 4.1 and Section 4.2, while recognizing that transfer to durable functional gain is inconsistent in the current literature (Section 4.3 and Section 6.3).

7. Do not assume that perturbation-based training is uniformly superior to conventional postural-control training. Where baseline trunk/postural control is markedly limited, conventional strengthening approaches may be a necessary precursor, consistent with the sequencing hypothesis proposed in Section 6.1.

**For both subtypes:**

8. Long-term musculoskeletal monitoring should be considered a standard component of any strategy involving sustained compensatory loading, given the elevated osteoarthritis and osteoporosis risks documented in adults with CP (Section 6.5). This applies with particular weight to the hemiplegic conservation strategy, the long-term joint health consequences of which remain untested.

In response to a request for greater transparency regarding effect magnitude, Table 4 summarizes the design, sample size, and principal quantitative findings of the key intervention and correlational studies discussed in Section 3, Section 4 and Section 6, including effect sizes, correlation coefficients, or hazard/odds ratios as reported by the original authors, so that readers can independently judge the magnitude of the effects underlying this review’s arguments.

### 7.2. Research Agenda

The recommendations above generate a correspondingly specific set of open questions, each traceable to an unresolved tension identified earlier in this review.

1. Test the reconciliation hypothesis for error augmentation and targeted-stimulation training (Section 6.1). Future split-belt, FES, and error-augmentation trials in hemiplegic CP should report post-treatment asymmetry values relative to a moderate-range target, not solely as a reduction from the baseline, to determine whether these protocols correct maladaptive escalation or eliminate adaptive compensation.
2. Test the sequencing hypothesis for diplegic postural training (Section 6.1). Trials directly comparing perturbation-based training preceded versus not preceded by baseline trunk-control strengthening are needed to determine whether Afzal et al.’s [58] finding reflects the genuine superiority of conventional training or an unaddressed prerequisite.
3. Prospective longitudinal musculoskeletal monitoring of children managed under a magnitude-bounded conservation strategy (Section 6.5). No existing study links a specific rehabilitation strategy (conservation vs. correction) to adult joint health outcomes; this is the single largest evidentiary gap bearing on the safety of the model’s central hemiplegic recommendation.
4. Independent replication of the neural independence mechanism (Section 6.4). The evidentiary concentration identified in Section 2.4 and Section 6.4 warrants replication in larger, more heterogeneous samples (varying lesion types, GMFCS level, spasticity grade) by research groups independent of the original cohort.
5. Systematic inter-limb asymmetry screening in diagnosed diplegic populations (Section 6.2). The prevalence and clinical significance of asymmetric bilateral spastic CP should be characterized in larger, prospectively designed cohorts to determine whether the ~32% figure from Gahukamble [55] generalizes and what threshold should trigger reclassification for strategy-selection purposes.
6. Prospective operationalization of the DRRF (Section 5.3). The four proposed domains require measurement protocols, pilot scoring, and threshold-setting before any claim of clinical utility can be made. This should be conducted as a dedicated methodological study, not as a byproduct of this review.
7. Identify the shared methodological limitations underlying inconsistent functional transfer (Section 6.3). Dosage, level of robotic/therapist assistance, and choice of outcome measure (impairment vs. participation-level) should be systematically varied across future robotic, split-belt, and perturbation trials in both subtypes to isolate the factor(s) that explain the recurring laboratory-to-function transfer gap.
8. The evidence base should be extended to more severely affected and non-ambulatory children (Section 6.6). Nearly all cited mechanistic and interventional evidence is drawn from GMFCS I–II and mild-to-moderate spasticity populations; the model’s applicability to GMFCS III–V and non-ambulatory presentations is currently untested and should not be assumed.

## 8. Conclusions

This review proposed and evidenced a trajectory-divergence model of gait-asymmetry rehabilitation in cerebral palsy. We argued that hemiplegic and diplegic CP represent opposing deviations from the normative developmental symmetry trajectory established in the companion review [1]: persistent, actively regulated compensatory asymmetry in hemiplegia, and pathological loss of adaptive motor variability in diplegia. Current rehabilitation practice treats gait symmetry index improvement as a near-universal marker of therapeutic success; we argued that this is misaligned with the distinction above for at least one of the two subtypes to which it is applied. Relative to the companion preprint [3], which established the descriptive trajectory-divergence model, the present review’s distinct contribution is prescriptive: the magnitude-boundary construct (Section 3.3), the Divergent Rehabilitation Response Framework (Section 5), the differentiated clinical recommendations with explicit certainty grading (Section 7), and the direct confrontation of counter-evidence, evidentiary concentration, and long-term safety uncertainty (Section 6) are original to this manuscript and are not present in [3].

The evidence assembled here supports a qualified, rather than an unconditional, version of this argument. For hemiplegic CP, compensatory asymmetry is mechanistically grounded in preserved, independently adaptable neural control of each limb (Section 3.1), functionally and energetically dissociable from the necessity of correction (Section 3.2), but bounded by magnitude: escalating asymmetry, not asymmetry as such, is associated with a deteriorating balance and locomotor capacity (Section 3.3). The clinical implication is therefore not to preserve asymmetry unconditionally but to prevent its escalation while resisting the reflexive correction of moderate, functionally compensatory patterns. For diplegic CP, the case for restoring adaptive motor variability is supported by convergent kinematic, neuromuscular, and interventional evidence (Section 4.1 and Section 4.2), but is conditional on confirming genuine bilateral rigidity—since a meaningful minority of diagnosed diplegic children present with their own inter-limb asymmetry (Section 4.3)—and is qualified by inconsistent functional transfer across the current perturbation- and robotics-based intervention literature (Section 4.3 and Section 6.3).

The Divergent Rehabilitation Response Framework (Section 5), offered as a provisional heuristic rather than a validated instrument, organizes this evidence by domain and explicitly states where the model is well supported (independent neural control for hemiplegia; variability restoration for diplegia) and where it is not (participation-level evidence for diplegia; any direct efficiency test of the conservation strategy for hemiplegia). Section 6 confronts the model’s principal counter-evidence directly: intervention studies in which reducing rather than preserving subtype-characteristic patterns produced functional gains, subtype heterogeneity that undermines a purely diagnosis-based strategy selection, a recurring pattern of laboratory-to-function transfer failure independent of subtype, a genuine concentration of the evidence base within a small number of research groups, and—most consequentially—the currently unresolved question of whether conserved compensatory asymmetry carries long-term musculoskeletal risk.

These limitations do not, in our assessment, undermine the case for reconsidering symmetrization as a universal rehabilitation goal in cerebral palsy; they define the terms on which that reconsideration should proceed. Accordingly, the contribution of this review is not a finished clinical protocol but a differentiated, evidence-anchored, and explicitly bounded alternative to the assumption that more symmetry is always better for the outcome. To date, this assumption has rarely been examined on its own terms in either hemiplegic or diplegic cerebral palsy.

## Figures and Tables

**Figure 1:**
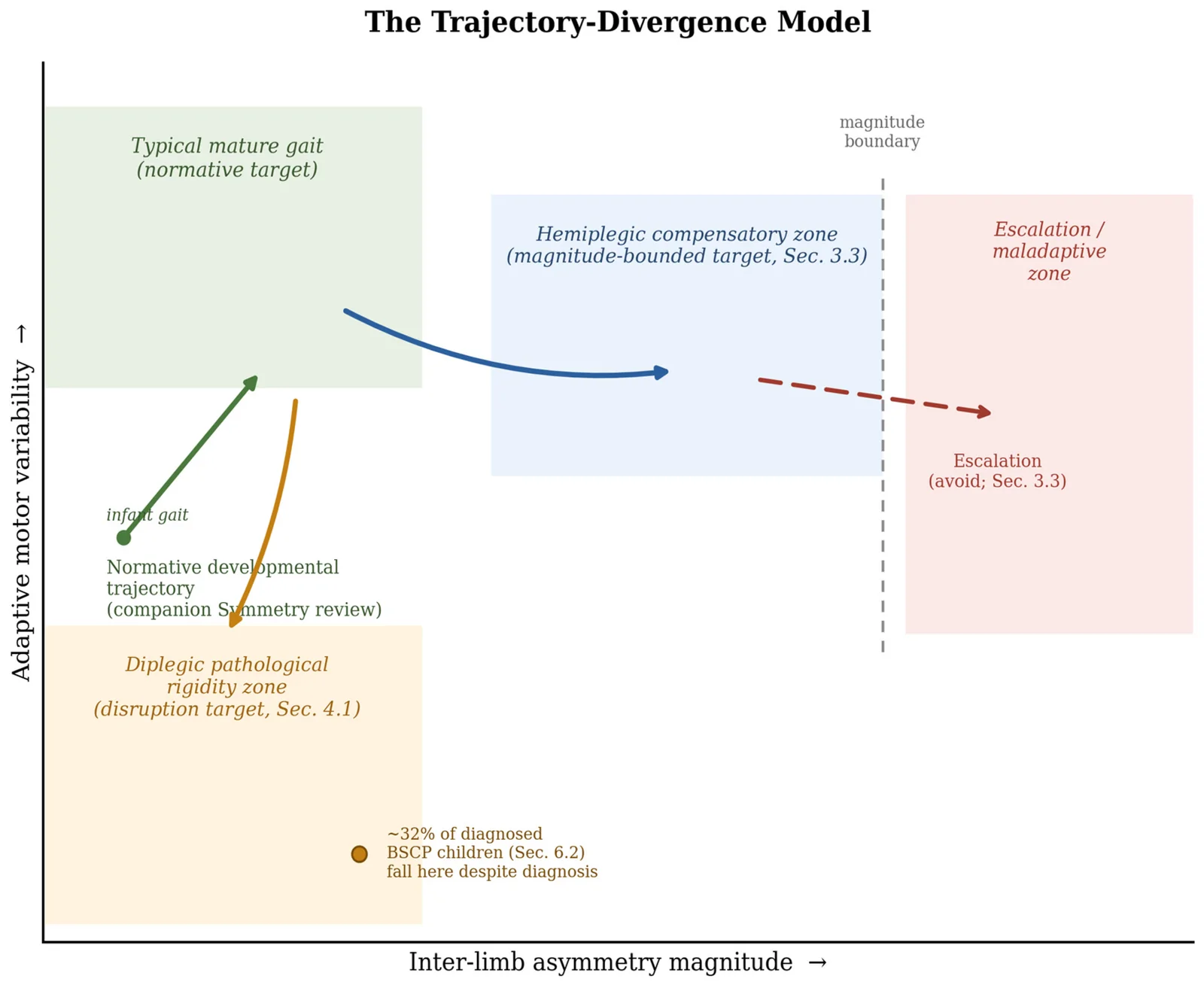
Conceptual scheme (not derived from empirical data or a statistical model) illustrating the trajectory-divergence hypothesis, plotted as inter-limb asymmetry magnitude (x-axis) against adaptive motor variability (y-axis). The normative developmental trajectory moves from infant gait toward the low-asymmetry, high-variability “typical mature gait” zone. Hemiplegic CP diverges into a magnitude-bounded compensatory zone (Section 3.3); diplegic CP diverges into a low-variability pathological rigidity zone (Section 4.1). The dashed line marks the magnitude boundary beyond which escalating asymmetry becomes associated with declining function (Section 3.3); the marked point indicates the subset of diagnosed bilateral spastic CP cases with clinically meaningful inter-limb asymmetry (Section 6.2).

**Figure 2:**
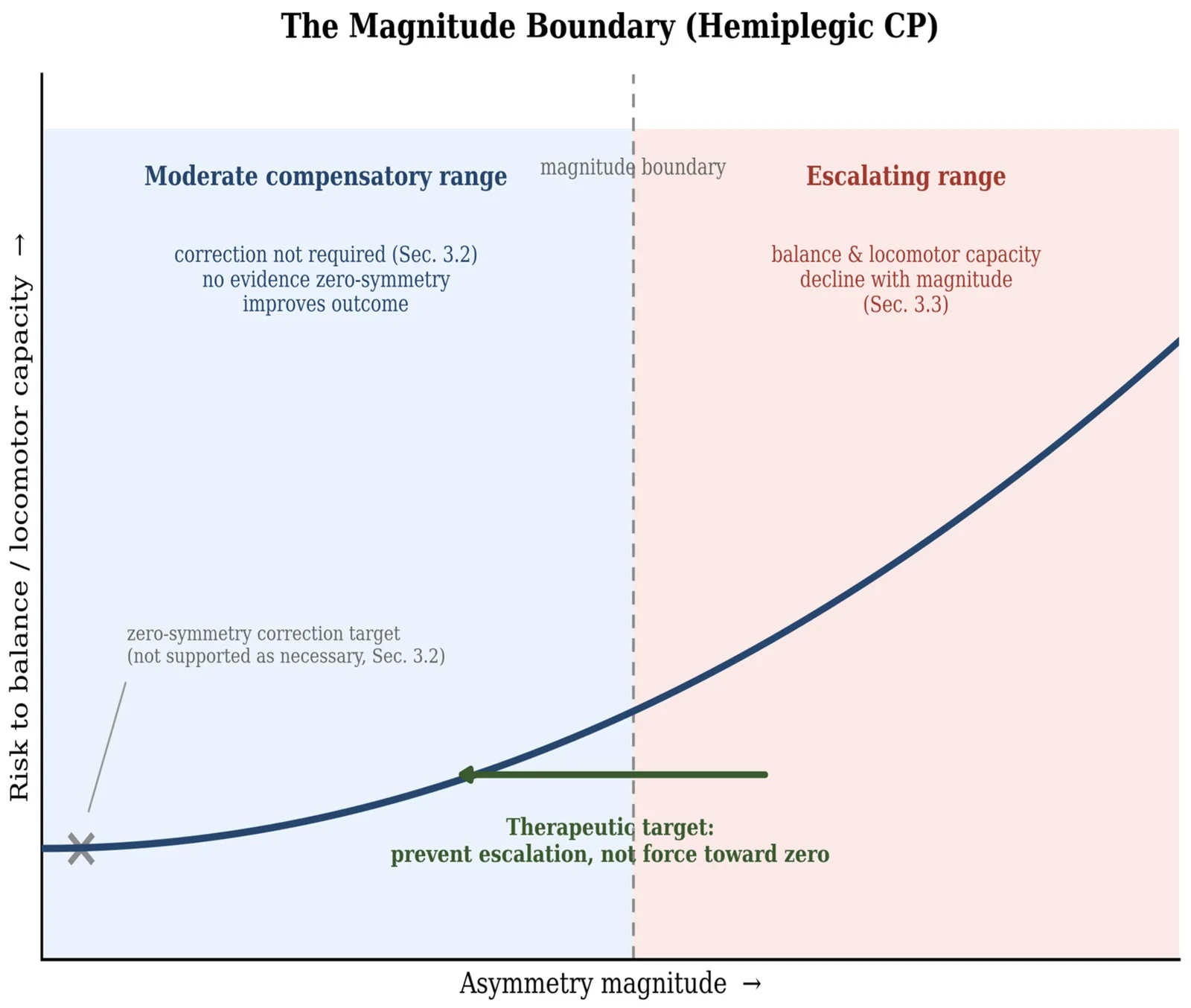
Hypothesized magnitude–risk relationship in hemiplegic CP (schematic, not derived from a validated threshold). The figure schematically illustrates the hypothesis that risk to balance and locomotor capacity increases with asymmetry magnitude (x-axis), consistent in direction with [25,27]; no study has identified a specific cut-off, and the dividing line shown is conceptual, not empirically derived. Within the lower range, correction toward zero symmetry is not supported as necessary (Section 3.2); at higher, escalating magnitudes, worsening function has been observed (Section 3.3). The therapeutic implication proposed is directional—avoiding further escalation—rather than a validated numerical target.

**Table 1:** Comparison of the hemiplegic and diplegic arms of the trajectory divergence model.

Dimension	Hemiplegic CP (Unilateral Spastic) (Section 3)	Diplegic CP (Bilateral Spastic) (Section 4)
Characteristic deviation	Persistent, actively regulated inter-limb asymmetry	Excessively rigid, over-coupled bilateral coordination (loss of adaptive variability)
Core mechanism	Preserved independent neural control of each limb [28,29]	Reduced muscle synergy complexity and inter-joint coordination variability [46,47]
Therapeutic target	Prevent asymmetry escalation; do not force zero symmetry (Section 3.3)	Restore adaptive, task-appropriate motor variability (Section 4.1 and Section 4.2)
Strongest supporting evidence	Independent-limb-control cohort [28,29]; energy/asymmetry-driver studies [26,41,42]	High-variability practice trial [49]; perturbation-training literature [50,51]
Principal boundary condition	Magnitude-dependent: escalating asymmetry associates with worse balance/function [25,27]	Subtype heterogeneity: ~32% of diagnosed cases show clinically meaningful asymmetry in a single, not-yet-replicated conference abstract [55] (preliminary estimate)
Principal counter-evidence	Error-augmentation split-belt training reduces asymmetry with functional benefit [24,45]	Trunk-control training outperforms perturbation training on all outcomes [58]
Evidentiary concentration risk	Mechanistic pillar rests on a single 10-child cohort (Section 6.4)	Conceptual premise and null-transfer evidence share an author group (Section 6.4)

**Table 2:** Divergent Rehabilitation Response Framework (DRRF): provisional domains and current evidentiary status by cerebral palsy subtype.

Domain	Hemiplegic CP (Unilateral Spastic)	Diplegic CP (Bilateral Spastic)
D1—Compensatory/variability efficiency	Directionally supported by energy-cost and asymmetry-driver studies [26,41,42]; no study directly tests preservation vs. correction on energy cost.	Best-evidenced domain for diplegia; high-variability practice improved gross motor performance and ADL independence [49], though from a single trial.
D2—Independent neural control/adaptive response	Most directly evidenced domain, resting on independent-limb-control findings [28,29]; subject to the evidentiary-concentration caveat (Section 6.4).	Supported by studies showing varied-force outperforms constant-force perturbation training [51,59,60]; may be self-limiting in children with low baseline variability.
D3—Participatory functionality	Substantially strengthened by longitudinal and participation-specific evidence [23,57,58,59]; conditional on the magnitude boundary (Section 3.3).	Weakest domain; only indirect evidence via ADL independence [49] and inconsistent functional-transfer findings (Section 4.3).
D4—Risk/safety	Reassuring in direction—asymmetric compensation appears protective [60,61,62]; qualified by magnitude-dependent balance findings [25].	Reassuring for perturbation-based training (reduced fall risk) [52]; qualified by trunk-control-training superiority findings [58].

**Note:** Domain scores are not quantified; this table summarizes the direction and qualification of available evidence, consistent with the DRRF’s status as a provisional heuristic (Section 5.1 and Section 5.3), not a validated scoring instrument.

**Table 3:** Differentiated clinical recommendations by subtype, with supporting rationale and qualitative certainty of evidence.

No.#	Recommendation	Certainty of Evidence	Applies to	Grounding Section
1	Do not treat residual gait asymmetry as a default correction target; prioritize functional gain over symmetry-index reduction	Low	Hemiplegic	Section 3.1 and Section 3.2
2	Monitor asymmetry magnitude, not merely its presence, to detect escalation	Moderate	Hemiplegic	Section 3.3
3	Where correction-oriented protocols are used, evaluate outcomes against a moderate-asymmetry target, not zero-symmetry	Low	Hemiplegic	Section 6.1
4	Restrict application to ambulatory, GMFCS I-II, mild-to-moderate spasticity pending further evidence	Not graded (precautionary)	Hemiplegic	Section 6.6
5	Confirm bilateral symmetry (screen for inter-limb asymmetry) before applying a disruption-based strategy	Very low	Diplegic	Section 6.2
6	Favor variability-restoring approaches over further static correction	Low	Diplegic	Section 4.1 and Section 4.2
7	Do not assume perturbation-based training is uniformly superior to conventional postural-control training	Low	Diplegic	Section 6.1 and Section 6.3
8	Treat long-term musculoskeletal monitoring as standard for any strategy involving sustained compensatory loading	Not graded (precautionary)	Both	Section 6.5

**Table 4:** Design, sample size, and key quantitative findings of the principal intervention and correlational studies underlying Section 3 and Section 4 and Section 6. Effect sizes, correlation coefficients, and hazard/odds ratios are reported as given by the original authors; entries marked “NS” indicate a non-significant finding. Full statistical detail (confidence intervals, exact *p*-values) should be verified against each primary source before clinical application. Abbreviations used in this table: RCT, randomized controlled trial; NS, non-significant; N, sample size; Sb-TW, split-belt treadmill walking; LoS, limit(s) of stability; D-LOSoverall, overall dynamic limit of stability; AI, asymmetry index; BOS, base of support; CoP, center of pressure; 6-MWT, 6-min walk test; TUDS, Timed Up and Down Stairs test; 10mSRT, 10-m shuttle run test; GDI, Gait Deviation Index; NDT, neurodevelopmental treatment; ADL, activities of daily living; FES, functional electrical stimulation; OA, osteoarthritis; OR, odds ratio; HR, hazard ratio; CI, confidence interval; TCMS, Trunk Control Measurement Scale; PBS, Pediatric Balance Scale; LEFS, Lower Extremity Functional Scale; GMFCS, Gross Motor Function Classification System.

Study [ref]	Design; N	Population	Key Quantitative Finding
Elnaggar et al. 2019 [21]	RCT; N = 24	Unilateral CP	↑ walking speed, stride length, step time (*p* < 0.05); weight-bearing symmetry index NS change
Elnaggar et al. 2023 [24]	RCT; N = 52 (26/26)	Unilateral CP, adolescents	Sb-TW vs. single-belt: greater step-length asymmetry reduction, improved LoS, locomotor capacity (6-MWT, TUDS, 10mSRT), *p* < 0.05
Elnaggar 2024 [25]	Cross-sectional; N = 85	Hemiplegic CP	AI-temporal vs. D-LOSoverall r = −0.437 (95% CI −0.245 to −0.592); AI-temporal vs. BOS r = 0.694 (95% CI 0.563–0.791)
Elnaggar & Elfakharany 2026 [27]	Longitudinal; N = 27; 2-year	Unilateral CP	Higher baseline asymmetry associated with slower functional improvement over 24 months (correlational)
Kwon & Ahn 2016 [49]	RCT; N not stated in abstract	Spastic diplegia	High-variability practice > standard NDT for gross motor performance and ADL independence, *p* < 0.05
Manikowska et al. [56]	Pre-post; small N	Bilateral spastic CP	No significant change in spatiotemporal parameters or GDI; limited symmetry improvement only
Phillips et al. [52]	Feasibility; small N	CP, mixed	Improved functional balance and increased time-to-contact (↓ fall risk), exact statistics not extracted from abstract
Afzal et al. 2025 [58]	RCT; small N (conference-linked)	CP, mixed	Trunk-control training > perturbation training on TCMS, PBS, LEFS, spinal alignment, all *p* < 0.05
El-Shamy & Abdelaal [67]	RCT	Hemiplegic CP	WalkAide FES reduced energy expenditure and improved gait pattern, *p* < 0.05
French et al. 2019 [68]	Matched cohort; large administrative database	Adults with CP	Adjusted OR 1.3–2.1 for hip/knee/poly/any OA (*p* < 0.001); OR 0.86 for hand OA (NS, *p* = 0.46)
O’Connell et al. 2019 [69]	Population-based cohort; N = 1705 CP/5115 controls	Adults with CP	Adjusted HR 6.19 (95% CI 3.37–11.39) osteoporosis; HR 1.54 (95% CI 1.17–2.02) osteoarthritis
Gagnat et al. 2023 [20]	Cross-sectional; N = 40 (71 observations)	Ambulant CP, GMFCS I-II	Gait deviation + height explained ~40% variance in energy cost; asymmetry NS additional contribution

## Data Availability

No new data were created in this study.

## Supplementary Materials

The following supporting information can be downloaded at: https://www.mdpi.com/article/10.3390/jfmk11040376/s1. Table S1: Full evidence table (71 entries—70 sources identified through the search strategy described in Section 2.2, plus the companion preprint [3], with thematic group mapping, DRRF domain tagging, and methodological notes on evidentiary concentration). Four further sources incorporated during peer review (refs. [4,5,6,20]; Section 2.2) were identified outside the declared search strategy and are accordingly not included in Table S1.

## Abbreviations

The following abbreviations are used in this manuscript:

CP	Cerebral palsy
PCC	Population–Concept–Context
JBI	Joanna Briggs Institute
GMFCS	Gross Motor Function Classification System
MAS	Modified Ashworth Scale
DRRF	Divergent Rehabilitation Response Framework
ADL	Activities of daily living
RCT	Randomized controlled trial
